# Supplementary material for: High prevalence of caesarean birth among mothers delivered at health facilities in Bahir Dar city, Amhara region, Ethiopia. A comparative study
Source: PLoS One. 2020 Apr 16;15(4):e0231631. doi: 10.1371/journal.pone.0231631 (PMC7162673; doi:10.1371/journal.pone.0231631)
Supplement: S1 File — (DOCX) [file pone.0231631.s001.docx]

## **English version questionnaire**

Structured English Version questionnaire and check list used to collect data to assess and compare caesarean birth and its associated factors among public and private health facility in Bahir Dar city, Amhara region, Ethiopia, 2019.

Participants identification code,__________ Name of health facility______________

**Part I: A Socio – demographic and economic background characteristics**

| **No** | **Questions & filter** | **Coding category** | **Skip to** |
| --- | --- | --- | --- |
| **No** | **Questions** | **Response** | **Skip to** |
| 101 | Age of the mother? | ___________years |  |
| 102 | Where is Your place of residence? | 1.urban  2.rural |  |
| 103 | What is your marital status? | 1.Single  2.Married  3.Divorced  4.Widowed |  |
| 104 | What is your religion? | 1. Orthodox 2. Catholic  3. Protestant 4. Muslim  5.Other(specify |  |
| 105 | What is your Ethnicity? | 1.Amhara 2. Oromo  3.Tigrie 4. Other (specify |  |
| 106 | What is your educational level? | 1. have no formal education  2. primary school (1-8)  3. secondary school (9-12)  4. Diploma & above---------------- |  |
| 107 | What is your occupation? | 1. House wife 2. Government employee 3. Private employee 4. Farmer 5. Merchant 6. Daily laborer 7. student 8. Other |  |
| 108 | What is your spouse level of education? | 1. have no formal education  2. primary school (1-8)  3. secondary school (9-12)  4. Diploma & above- |  |
| 109 | What is your spouse occupation? | 1. Civil servant 2. Self employed 3. Farmer 4. Merchant   5. Other(specify)(NGO) |  |

**Household Wealth index related questions**

1. **Urban resident**

| No | Questions | Response | Skip |
| --- | --- | --- | --- |
| 110 | How much the average monthly family income | __________________birr |  |
| 111 | Do you have your own house? | 0)No  1)Yes |  |
| 112 | What is the roof of the main house? | 1.Corrugated iron sheet  2.grass  3.Other specify ----------- |  |
| 113 | What is the wall of the main house? | 1.Mud  2.Cement  3.Bricks  4.Other specify -------- |  |
| 114 | What is the floor of the main house? | 1.Soil  2.Cement  3.Ceramic  4.Other specify ---------- |  |
| 115 | Have you electricity for light source | 0)No 1)Yes |  |
| 116 | Have you radio in your family | 0)No 1)Yes |  |
| 117 | Have you television | 0)No 1)Yes |  |
| 118 | Have any one mobile in your family member | 0)No 1)Yes |  |
| 119 | Availability of non-mobile telephone | 0)No 1)Yes |  |
| 120 | Have you refrigerator in your house | 0)No 1)Yes |  |
| 121 | Have you chair in your house | 0)No 1)Yes |  |
| 122 | Have you table in your house | 0)No 1)Yes |  |
| 123 | Have you bed with cotton/sponge/spring matters in your house | 0)No 1)Yes |  |
| 124 | Have you electric cooking materials in your house | 0)No 1) Yes |  |
|  |  |  |  |

1. **Rural resident**

| 125 | Do you have your own house? | 0.No 1. Yes   1. 1.   1. አዎ |
| --- | --- | --- |
| 126 | Types of house? | 0.grass 1. ron sheet |
| 127 | Have you radio in your family? | 0)No 1)Yes |
| 128 | Have you television ? | 0)No 1)Yes |
| 129 | Have any one mobile in your family member | 0)No 1)Yes |
| 120 | Have you table in your house | 0)No 1)Yes |
| 121 | Have you bed with cotton/sponge/spring matters in your house? | 0)No 1)Yes |
| 122 | Have you solar light source in the house? | 00)No 1)Yes |
| 123 | Have you land for farming? | 0)No 1) Yes |
| 124 | Your own land by local measurement  Renting by local measurement | 1. ----gemmed in amharic /hectare 2. ----gemmed in amharic/ hectare |
|  | **How much the year-based farm product?** | |
| 125 | Dagusa in Amharic | ____________ kuntal in amharic |
| 126 | Teff | _____________kuntal in amharic |
| 127 | Maze | _____________kuntal in amharic |
| 128 | Shimbra in amharic | ____________kuntal in amharic |
| 129 | Rouse | ___________kuntal in amharic |
|  | **Number and types of animal at house hold level** | |
| 130 | Horn animal (ox, cow) | ____________ In number |
| 131 | Noe horn animal (house, donkey) | _____________ In number |
| 132 | Sheep | _____________ In number |
| 133 | Goat | _____________ In number |
| 134 | hen | ____________ In number |
| 135 | Hany bee( by kefo in Amharic) | _____________ In number |

**Part II- Past obstetrics history**

| No | Questions | Coding category | Skip to |
| --- | --- | --- | --- |
| 201 | How many times have you ever been pregnant? | ______in number | If 0 skip to part III |
| 202 | Have you ever had stillbirth (after 7moths of GA)? | 0)No  1)Yes | If 0 skip to Q 204 |
| 203 | If yes in Q 202 How many times ever had stillbirth? | ______in number |  |
| 204 | Have you ever had abortion (before 7moths of GA)? | 0)No  1)Yes | If 0 skip to Q 206 |
| 205 | If yes in Q 204 How many times had abortion? | ______in number |  |
| 206 | Have you alive children? | 0)No  1)Yes | If 0 skip to Q 208 |
| 207 | If yes in question 206, How many a live child do you have? | ______in number |  |
| 208 | How many times you give birth | ______in number |  |
| 209 | Do you have a history of previously diagnosed infertility problem | 0)No  1)Yes |  |
| 210 | What was your previous mode of delivery? | 1.Vaginaldelivery _________number  2.Caesarean section _________number |  |

**Part III- Current obstetrics history**

| **No** | **Questions** | **Coding category** | **Skip to** |
| --- | --- | --- | --- |
| 301 | Did you attend antenatal care in the current pregnancy? | 0)No  1)Yes | If No, skip to question 06 |
| 302 | For how many times did you have ANC follow up for current pregnancy? | _________times |  |
| 303 | Have you informed the number of the fetus before delivery? | 0)No  1)Yes |  |
| 304 | If yes in Q303 how many fetus? | 1. Singleton 2. Twin 3. Other (specify ) |  |
| 305 | How to come this health facility? | 1.referred from health center  2. referred from health post  3. referred from private facility’s  4.directly come  5.referred from district hospital |  |
| 306 | How was labor started | 1. Spontaneously 2. Induced/Elective CS |  |
| 307 | What was the mode of delivery in current pregnancy? | 1. Vaginal delivery  2. caesarean section delivery |  |

**PART IV. Checklist for client chart review**

1. **For all delivered mothers**

| No | **Questions** | **Coding category** | **Skip to** |
| --- | --- | --- | --- |
| 401 | What was the gestational age at current delivery? | _______ in weeks |  |
| 402 | Onset of labor | 1)Spontaneously  2)Induced/elective Cs |  |
| 403 | Maternal complication identified during pregnancy? | _______ |  |
| 1. **For only women gave caesarean birth** | | | |
| 405 | Current cesarean section of delivery | 1.Primary (for the first time)  2.Repeat (in number)_________ |  |
| 406 | What is the type of caesarean section performed based on urgency? | 1) Emergency  2) Elective |  |

| 407 | What was the primary indication for the current caesarean section? | ___________________ |  |
| --- | --- | --- | --- |
| 408 | Status of women after operation | 1. Alive 2. Dead |  |

1. **Neonatal outcome for all Newborn**

| No | Questions | Coding category | Skip to |
| --- | --- | --- | --- |
| 409 | Fetal presentation | ­­­­­­­­­­­­­­­­­­­­­­­--------------- |  |
| 410 | The number of newborns delivered | ­­­­­­­­­­­­­­__________in number |  |
| 411 | Fetal outcome | 1. Alive 2. Dead (still birth) |  |

**የአማረኛ መጠይቅ**

የአማረኛ መተይቅ ባህርዳር ከተማ በመንግስት እና በግል ጤና ተቋማት ዉስጥ በኦፕሬሽን የሚወልዱ እናቶችን መጥን ለማወቅ፣ በገልና መንግስት ተቋም በኦፕሬሽን የሚወልዱትን መጠን ለመወዳደር ኣና ምክንያቶችን ለማዎቅ የተዘጋጀ መጠይቅ

የተሳታፊዋ መለያ ኮድ-------------የማዋለድ አገልግሎት የተሰጠበት ጤና ተቋም ስም--------------

**ክፍል 1 ፡ማህበራዊ መረጃዎችን በተመለከተ የቀረበ መጠይቅ**

| **ተ.ቁ** | **ጥያቄ** | **የመልስ አማራጭና መለያ ኮድ** | **ይዘለል** |
| --- | --- | --- | --- |
| 101 | እድሜዎ ስንት ነዉ ? | ­­­­­­­­­­­­­­­­-------------ዓመት |  |
| 102 | የመኖሪያ አድራሻዎ የት ነዉ? | 1. ከተማ 2. ገጠር |  |
| 103 | የጋብቻዎ ሁኔታ ምንድን ነው ? | 1. ያላገባች 2. ያገባች 3. ከባሏየተፋታች 4. ባሏየሞተባት |  |
| 104 | ሀይማኖትዎ ምንድን ነው? | 1. ኦርቶዶክስ  2. ካቶሊክ  3. ፕሮቲስታን  4. ሙስሊም  5. ሌላከሆነ ( ይገለጽ)---------------- |  |
| 105 | ብሄርዎ ምንድን ነዉ? | 1. አማራ  2. ኦሮሞ  3. ትግሬ  4. ሌላከሆነ ( ይገለጽ) |  |
| 106 | የትምህርት ደረጃወት ምን ያህል ነዉ? | 1.መደበኛትምህርት ያልተመሩ  2. የአንደኛ ደረጃ (1-8 ክፍል)  3. ሁለተኛ ደረጃ(9-12 ክፍል)  4. ዲፕሎማ እና ከዚያ በላይ |  |
| 107 | ሥራዎት ምንድን ነዉ? | 1. የቤትእመቤት 2. የመንግስትሠራተኛ 3. የግልሥራሠራተኛ. 4. ግብርና 5. ነጋዴ 6. የቀን ሠራተኛ 7. ተማሪ 8. ሌላ(ይገለፅ)____________ |  |
| 108 | የባለቤትዎ የትምህርት ደረጃ ምን ያህል ነዉ? | 1. መደበኛትምህርት ያልተመሩ  2. የአንደኛ ደረጃ (1-8 ክፍል)  3. ሁለተኛ ደረጃ(9-12 ክፍል)  4. ዲፕሎማ እና ከዚያ በላይ |  |
| 109 | የባለቤትዎ ሥራ ምንድን ነዉ? | 1. የመንግስት ሠራተኛ 2. የግል ሠራተኛ. 3. ግብርና 4. ነጋዴ 5. የቀንሠራተኛ 6. ተማሪ 7. ሌላ(ይገለፅ)____________ |  |
|  |  |  |  |

**የቤተሰብ የሃብትመጠን መለኪያ ጥያቄዎች**

**ሀ. ለከተማ ነዋሪዎች ብቻ የሚጠየቅ**

| **ተ.ቁ** | **ጥያቄ** | **የመልስ አማራጭና መለያ ኮድ** | **ይዘለል** |
| --- | --- | --- | --- |
| 110 | የቤተሰብዎ አማካኝ የወር ገቢ ምን ያህል ነዉ? | ----------------ብር |  |
| 111 | የግልዎት የመኖሪያ ቤት አለዎት? | 0. የለኝም 1. አዎ |  |
| 112 | የዋናዉ ቤትዎ ጣሪያ የተሰራዉ ከምንድን ነዉ? | 1. ከቆርቆሮ 2. ከሳር 3. ከሌላ(ይገለፅ)________ |  |
| 113 | የዋናዉ ቤትዎ ግድግዳ የተሰራዉ ከምንድን ነዉ? | 1. ከጭቃ 2. ከስሚንቶ 3. ከጡብ 4. ከሌላ(ይገለፅ)________ |  |
| 114 | የዋናዉ ቤትዎ ወለል የተሰራዉ ከምንድን ነዉ? | 1. ከአፈር 2. ከስሚንቶ 3. ከሴራሚክ 4. ከሌላ(ይገለፅ)_________ |  |
| 115 | የኤሌክትሪክ መብራት አገልግሎት ያገኛሉ? | 0. የለም 1. አዎ |  |
| 116 | ቤትዎ ዉስጥ ሬዲዮ አለወት? | 0. የለም 1. አዎ |  |
| 117 | ቤትዎ ዉስጥ ቴሌቪዥን አለወት? | 1. የለም 1. አዎ |  |
| 118 | ከቤተሰብ ውስጥ ተንቀሳቃሽ ስልክ ያለው አለ? | 1. የለም 1. አዎ |  |
| 119 | የመኖሪያ ቤት ስልክ አለዎት? | 1. የለም 1.አዎ |  |
| 120 | ቤትዎ ዉስጥ ማቀዝቀዣ/refrigerator አለወት? | 1. የለም 1. አዎ |  |
| 121 | ቤትዎ ዉስጥ ወንበር አለ ? | 1. የለም 1. አዎ |  |
| 122 | ቤትዎ ዉስጥ ጠረፔዛ አለ? | 1. የለም 1. አዎ |  |
| 123 | ቤትዎ ዉስጥ አልጋ ከነፍራሹ (የጥጥ፣ስፖነጅ፣እስፕሪንግ) አለ? | 1. የለም 1. አዎ |  |
| 124 | ቤትዎ ዉስጥ የኤሌክትሪክ ምጣድ አለ? | 1. የለም 1. አዎ |  |
|  |  |  |  |

**ለ. ለገጠር ነዋሪዎች ብቻ የሚጠየቅ**

| 125 | መኖሪያ ቤቱ የራስዎ ነው? | 0.አደለም 1. አወ   1. 1.   1. አዎ |
| --- | --- | --- |
| 126 | የቤቱ አይነት? | 0.ሳር 1. ቆርቆሮ |
| 127 | ሬዴዮ | 0. የለም 1. አለ  1. አዎ |
| 128 | ከብተሰብዎ መካከል ሞባይል ያለው አለ? | 0. የለም 1.አለ  1. አዎ |
| 129 | ወንበር? | 0. የለም 1 አለ  1. አዎ |
| 120 | ጠረንፔዛ? | 0. የለም 1. አለ  1. አዎ |
| 121 | አልጋእናከጥጥ፣እስፖንጅየተሰራፍራሽ | 0. የለም 1. አለ  1. አዎ |
| 122 | ሶላር መብራት | 0. የለም 1. አለ  1. አዎ |
| 123 | የሚታረስ መሬት አላችሁ | 0. የለም 1. አለ  1. አዎ |
| 124 | በግል (አካባቢውመለኪያ)  የክራይ (አካባቢውመለኪያ) | 1. ------------ በገመድ/ሄክታር 2. ------------ በገመድ/ሄክታር |
|  | **አመታዊ የምርት ገቢ መጠን በኩንታል ስንት ነው** | |
| 125 | ዳጉሳ | ____________ኩንታል |
| 126 | ጤፍ | ______________ኩንታል |
| 127 | በቆሎ | ______________ኩንታል |
| 128 | ሽንብራ | ______________ኩንታል |
| 129 | ሩዝ | _____________ኩንታል |
|  | **የእንስሳት ሃብት መጠን እና አይነት በቤተሰብ ደረጃ** | |
| 130 | በሬ፣ላም፣ጥጃ | በቁጥር____________ |
| 131 | ፈረስ፣አህያ | በቁጥር_____________ |
| 132 | በግ | በቁጥር_____________ |
| 133 | ፍየል | በቁጥር_____________ |
| 134 | ደሮ | በቁጥር____________ |
| 135 | የንብቀፎ | በቁጥር_____________ |

**ክፍል 2፡የድሮ እርግዝና ታሪክን የተመለከተ መጠይቅ**

|  | **ጥያቄ** | **ቁጥር** | **ዝለል** |
| --- | --- | --- | --- |
| 201 | ከዚህ በፊት ምን ያህል ጊዜ አርግዘው ነበር? | ---------------በቁጥር | 0 ከሆነወደ 208 ዝለል/ይ |
| 202 | ከ7 ወር እርግዝና በሁላ ሞቶ የተወለደ ልጅ አጋጥሞዎት ያዉቃል? | 1. የለም 2. አወ | የለም ከሆነ ወደ 204 ዝለል/ይ |
| 203 | ለ202 ጥያቄመልሱ አወከሆነ ምንያህል ልጆች ከ 7 ወርእርግዝና በሁላ ሞቶብዎታል? | ____________በቁጥር |  |
| 204 | ከዚህ በፊት ውርጃ አጋጥሞወት ያዉቃል( (ከ 7 ወራቶችበፊት)? | 1. የለም 2. አወ | የለም ከሆነ ወደ 206 ዝለል/ይ |
| 205 | ለ 204 ጥያቄ መልሱ አወ ከሆነ ለምን ያህል ጊዜ ውርጃ ፈጽመዋል/አጋጥምዎታል( (ከ 7 ወራቶችበፊት)? | ____________በቁጥር |  |
| 206 | በህይወት ያለ ልጅ አለዎት? | 1. የለም 1. አወ | የለም ከሆነወደ 208 ዝለል/ይ |
| 207 | ለ206 ጥያቄ መልሱ አወ ከሆነ ስንት በህይወት ያሉ ልጆች አሉዎት? | ____________በቁጥር |  |
| 208 | ከዚህ በፊት በሐኪም ልጅ የመዉለድ ችግር አለብዎ ተብለው ያወቃሉ? | 1. የለም 2. አወ |  |
| 209 | ከአሁኑ በፊት ምን ያህል ጊዜ ወልደዋል? | ____________በቁጥር |  |
| 210 | ከዚህ በፊት የወለዱት በምን መልኩ ነበር? | 1. በማህጸን-------በቁጥር 2. በኦፕራሲዮን---------በቁጥር | በማህጸንከሆነወደክፍል 4 ዝለል/ይ |

**ክፍል 2 ፡የአሁኑ እርግዝና ታሪክ መረጃ**

| ተ.ቁ | ጥያቄ | ኮድ | ዝለል |
| --- | --- | --- | --- |
| 301 | በአሁኑ እርግዝናዎት የቅድመ ወሊድ ክትትል አደረርገው ነበር? | 1. አላደረግሁም 2. አዎ | 1. አላደረግሁም   ከሆነ ወደ 305ኛጥያቄዝለል/ይ |
| 302 | በአሁኑ እርግዝናዎት ምን ያህል ጊዜ የቅድመ ወሊድ ክትትል አድርገዋል? | ________በቁጥር |  |
| 303 | በቅድመ ወሊድ ክትትልዎ ወቅት በማህፀንዎ ወስጥ ስለነበረዉ የፅንስ ቁጥር መረጃ ተሰጥቶዎ ነበር? | 1. የለም 2. አዎ |  |
| 304 | ለ303 ጥያቄመልሱ አወከሆነ ምን ያህል የፅንስ ቁጥር ተነገርዎ? | 1. አንድ 2. ሁለት 3. ሌላቁጥርከሆነይገለፅ------ |  |
| 305 | ወደ ወለዱበት ጤና ተቋሙ ለወሊድ እንዴት ሊመጡ ቻሉ? | 1. ከጤናጣቢያተልኬ/ ሪፈርተብየ 2. ከጤና ኬላ ተልኬ/ ሪፈር ተብየ 3. ከግልጤና ተቋም ተልኬ/ሪፈርተብየ 4. በራሴ ምርጫ |  |
| 306 | የወሊድ ምጡ እንዴት ጀመረዎ? | 1. በራሱ ጊዜ 2. በምጥ መድሀኒት 3. ምጥ አልጀመረኝም |  |
| 307 | ያሁኑን ልጅ እንዴት ነው የወለዱት? | 1. በማህፀን 2. በቀዶ ጥገና |  |
