# Supplementary material for: High prevalence of caesarean birth among mothers delivered at health facilities in Bahir Dar city, Amhara region, Ethiopia. A comparative study
Source: PLoS One. 2020 Apr 16;15(4):e0231631. doi: 10.1371/journal.pone.0231631 (PMC7162673; doi:10.1371/journal.pone.0231631)
Supplement: S1 Table — (DOCX) [file pone.0231631.s003.docx]

| Factors | Public health facilities(n=356) | | | | Private health facilities(n=352) | | | |
| --- | --- | --- | --- | --- | --- | --- | --- | --- |
|  | **CS** | | **COR (95%CI)** | **AOR (95%CI)** | **CS** | | **COR (95%CI)** | **AOR (95%CI)** |
|  | **No** | **Yes** |  |  | **No** | **Yes** |  |  |
| Fetal presentation | | | | | | | | |
| Cephalic | 241 | 83 | 1 | 1 | 149 | 184 | 1 |  |
| Breech | 17 | 15 | 2.56(1.22,5.3) | **3.64(1.49,8.89)** | 5 | 14 | 2.26(0.79,6.43) |  |
| Spouse education | | | | | | | | |
| No education | 84 | 15 | 1 | 1 | 2 | 1 | 1 | 1 |
| Primary | 38 | 15 | 2.21(0.98,4.97) |  | 7 | 5 | 1.42(0.1,20.43) |  |
| Secondary | 53 | 30 | 3.17(1.56,6.43) |  | 32 | 27 | 1.68(0.14,19.64) |  |
| Collage &above | 79 | 32 | 2.26(1.14,4.50) |  | 113 | 164 | 2.9(0.26,32.39) |  |
| Residence | | | | | | | | |
| Rural | 55 | 7 | 1 | 1 |  |  |  |  |
| Urban | 203 | 91 | 3.52(1.54,8.0) | **6.54(2.59,16.48)** | 154 | 198 |  |  |
| Referral status | | | | | | | | |
| Not referred | 153 | 44 | 1 | 1 | 154 | 198 |  |  |
| Referred | 105 | 54 | 1.78(1.11,2.85) | **2.44(1.46,4.08)** |  |  |  |  |
| Age of mother | | | | | | | | |
| 15-24 | 82 | 25 | 0.73(0.32,1.65) |  | 35 | 22 | 0.45(0.21,0.96) | **0.2(0.07,0.52)** |
| 25-34 | 147 | 61 | 1.00(0.48,2.09) |  | 95 | 143 | 1.09(0.6,1.96) | 0.51(0.24,1.08) |
| >35 | 29 | 12 | 1 |  | 24 | 33 | 1 | 1 |
| Maternal Education | | | | | | | | |
| No formal education | 84 | 23 | 1 |  | 2 | 1 | 1 | 1 |
| Primary school | 62 | 27 | 1.59(0.83,3.0) |  | 26 | 13 | 1.0(0.08,12.1) |  |
| Secondary school | 63 | 29 | 1.68(0.88,3.18) |  | 56 | 39 | 1.39(0.12,15.9) |  |
| College &above | 49 | 19 | 1.41(0.7,2.86) |  | 70 | 145 | 4.14(0.36,46.41) |  |
| Occupation | | | | | | | | |
| House wife | 180 | 68 | 1 |  | 87 | 65 | 1 | 1 |
| Government employee | 32 | 12 | 0.99(0.48,2.03) |  | 53 | 105 | 2.65(1.67,4.2) | **2.28(1.39,3.75)** |
| Self-employee | 46 | 18 | 1.03(0.56,1.91) |  | 13 | 27 | 2.78(1.33,5.8) | **3.73(1.62,8.59)** |
| NGO |  |  |  |  | 1 | 1 | 1.33(0.08,21.8) | 1.41(0.08,24.9) |
| Gravid | | | | | | | | |
| One | 45 | 106 | 1.3(0.7,2.63) |  | 111 | 73 | 3.34(1.11,10.02) |  |
| Two | 23 | 65 | 1.08(0.52,2.27) |  | 60 | 43 | 3.07(0.999.47) |  |
| Three | 14 | 38 | 1.12(0.49,2.59) |  | 22 | 27 | 1.79(0.54,5.93) |  |
| Four & above | 16 | 49 | 1 |  | 5 | 11 | 1 | 1 |
| Parity | | | | | | | | |
| One | 48 | 108 | 1.36(0.7,2.63) |  | 114 | 73 | 3.43(1.14,10.29) | **6.79(2.02,22.7)** |
| Two | 22 | 63 | 1.07(0.5,2.25) |  | 58 | 43 | 2.96(0.96,9.17) | **3.88(1.15,13.0)** |
| Three | 12 | 38 | 0.96(0.41,2.28) |  | 21 | 27 | 1.71(0.57,5.68) | 1.77(0.49,6.36) |
| Four &above | 16 | 49 | 1 |  | 5 | 11 | 1 | 1 |
| Gestational age | | | | | | | | |
| <37 | 24 | 7 | 1 |  | 1 | 2 | 1 | 1 |
| 37-42 | 233 | 88 | 1.29(0.53,3.11) |  | 151 | 192 | 0.63(0.57,7.07) |  |
| >42 | 1 | 3 | 10.2(0.91,115) |  | 2 | 4 | 1.0(0.05,18.9) |  |
| Wealth index | | | | | | | | |
| Lowest | 55 | 20 | 1 |  | 6 | 3 | 1 | 1 |
| Second | 51 | 17 | 0.91(0.43,1.94) |  | 55 | 56 | 2.03(0.48,8.55) | 1.71(0.37,7.89) |
| Medium | 43 | 23 | 1.47(0.71,3.02) |  | 32 | 32 | 1.82(0.42,7.92) | 1.62(0.33,7.78) |
| Fourth | 75 | 28 | 1.02(0.52,2.0) |  | 42 | 61 | 2.9(0.68,12.26) | 2.32(0.5,10.8) |
| Highest | 33 | 10 | 0.83(0.34,1.99) |  | 16 | 46 | 5.75(1.28,25.72) | **5.39(1.08,26.8)** |
